# Reclamation of Fishery Processing Waste: A Mini-Review

**DOI:** 10.3390/molecules24122234

**Published:** 2019-06-14

**Authors:** Chi-Hao Wang, Chien Thang Doan, Van Bon Nguyen, Anh Dzung Nguyen, San-Lang Wang

**Affiliations:** 1 Department of Chemistry, Tamkang University, New Taipei City 25137, Taiwan; 803160018@s03.tku.edu.tw (C.-H.W.); doanthng@gmail.com (C.T.D.); 2 Department of Science and Technology, Tay Nguyen University, Buon Ma Thuot 630000, Vietnam; bondhtn@gmail.com; 3 Institute of Biotechnology and Environment, Tay Nguyen University, Buon Ma Thuot 630000, Vietnam; nadzungtaynguyenuni@yahoo.com.vn; 4 Life Science Development Center, Tamkang University, New Taipei City 25137, Taiwan

**Keywords:** bioactive compounds, food byproducts, green extraction technologies, bioconversion

## Abstract

Seafood such as fish, shellfish, and squid are a unique source of nutrients. However, many marine processing byproducts, such as viscera, shells, heads, and bones, are discarded, even though they are rich sources of structurally diverse bioactive nitrogenous components. Based on emerging evidence of their potential health benefits, these components show significant promise as functional food ingredients. Fish waste components contain significant levels of high-quality protein, which represents a source for biofunctional peptide mining. The chitin contained in shrimp shells, crab shells, and squid pens may also be of value. The components produced by bioconversion are reported to have antioxidative, antimicrobial, anticancer, antihypertensive, antidiabetic, and anticoagulant activities. This review provides an overview of the extraordinary potential of processing fish and chitin-containing seafood byproducts via chemical procedures, enzymatic and fermentation technologies, and chemical modifications, as well as their applications.

## 1. Introduction

The amount of shrimp and crab waste from shellfish processing has undergone a dramatic increase recently. In addition to edible parts, the amount of chitin-containing waste can be as high as 60%–80% of the biomass [1,2]. Squid processing also generates a large amount of byproducts. These represent 35% of the total mass caught and include the head, viscera, skin, and bones [3]. To offset environmental pollution and disposal problems, marine byproducts are used to produce silage, meal, and sauces. They are also used in the production of value-added products, such as proteins, hydrolysates, bioactive peptides, collagen, gelatin, and chitin [1,2].

Numerous studies have demonstrated that byproducts from fish, shellfish, and squid processing are suitable for human consumption, animal food, and other applications with high market value [1,2,3]. Indeed, these marine byproducts are a source of interest for their collagen, peptide, polyunsaturated fatty acid, and chitin content. This review provides an overview of the extraordinary potential of fish processing byproducts and their applications.

## 2. Use of Fish Processing Byproducts

When fish are processed, heads, frames, viscera, scales, and skins are the major byproducts (Table 1) [4,5,6,7,8,9,10,11,12,13,14,15,16,17,18,19,20,21,22,23,24,25,26,27,28,29,30,31,32]. Among these fish processing byproducts, collagen is the main structural protein in scales and skin, representing up to 70% of their dry weight [32]. Fish viscera is also a potential source of lipids, native proteins, and hydrolysates [33]. The recovered products may possess functional and bioactive properties that are important to the food, agricultural, cosmetic, pharmaceutical, and nutraceutical industries [32,33].

There are three ways to reclaim bioactive materials from fish processing byproducts: (1) Chemical and/or physical methods that treat fish byproducts with chemicals and/or physical agents; (2) enzymatic methods that use enzymes (especially commercial proteases) to hydrolyze byproducts; and (3) microbial fermentation that uses microorganisms as the source of enzymes to obtain bioactive materials. Of the three, the enzymatic method seems best at recovering bioactive materials from fish heads, frames, viscera, scales, and skins [32,33].

### 2.1. Chemical and/or Physical Procedures

#### 2.1.1. Head and Frame

Microwaves and/or chemical treatments have been used to produce fish protein hydrolysates (FPH) from the heads and frames of kingfish [4]. Microwave intensification can significantly increase the production yields of enzymatic processes from 42% to 63%. It also increases the production yields of chemical processes from 87% to 98%. The chemical process and the microwave-intensified chemical process produce FPH with a low oil-binding capacity (8.66 and 6.25 g oil/g FPH, respectively), whereas the microwave-intensified enzymatic process produces FPH with the highest oil-binding capacity (16.4 g oil/g FPH). Due to the high content of histamine, the FPH produced by these processes demonstrates that the maximum proportion of FPH that can be safely used in food formulation is 10% [4].

Bones from carp and redfish have been used to produce collagen peptides via acid/alkali hydrolysis [18,19].

#### 2.1.2. Viscera

Blanco et al. reported the isolation and partial characterization of trypsin from the pancreas of the small-spotted catshark (*Scyliorhinus canicula*). Fish viscera have been documented to be an important source of enzymes that can be used in several industrial applications. In one study, trypsin was purified from the pancreas of *S. canicula* by ammonium sulfate precipitation and soybean trypsin inhibitor Sepharose 4B affinity chromatography [17]. The SDS-PAGE (sodium dodecyl sulfate-polyacrylamide gel electrophoresis) results showed that the isolated trypsin had a molecular weight of approximately 28 kDa and an approximate isoelectric point value of 5.5. The optimum pH and temperature for activity were 8.0 and 55 °C, respectively [17]. 

Fish oil can be extracted from fish viscera by various processes, including rendering, pressing, microwave-assisted extraction, supercritical fluid extraction, solvent extraction, autolysis, and enzymatic hydrolysis [34]. The wet rendering extraction method has been used to extract fish oil from tilapia and mackerel viscera. The oil yield obtained from tilapia viscera is about 20% which is 7% higher than that obtained from mackerel viscera [35].

Studies have examined the extraction of oil from tuna byproducts using the wet press and enzymatic extraction methods. The quantitative comparison and yield of the extracted oil by the wet press and enzymatic extraction methods have revealed the suitability of both methods for oil extraction in terms of quantity [36].

Based on reviewed scientific papers, the most promising green extraction method is oil extraction using supercritical CO_2_; the other methods described are still being developed [34].

#### 2.1.3. Scales

Carp and redfish scales have been treated by acid/alkali procedures to produce collagen peptides [18]. To make more effective use of underutilized resources, collagen from redfish scales [18,19] and croceine croaker [28] has been isolated with acetic acid and characterized for its potential in commercial applications [19].

The scales of the Nile tilapia have been used for metallic ion removal following acid demineralization and a basic deproteinization treatment to modify the organic/inorganic matter ratios [20]. Two main fractions, the organic fraction (protein) and the inorganic fraction (mainly composed of hydroxyapatite), of Nile tilapia scales have been studied for their adsorptive capacity. When the pure organic and inorganic parts of the fish scales are used in adsorption experiments, the inorganic part has a 75% higher removal capacity than the organic fraction. Adsorption experiments using fish scales with different organic or inorganic fractions have shown a synergistic effect on the equilibrium amount of metallic ions adsorbed. The main mechanism for metallic ion adsorption by fish scales is suggested by the ion-exchange reaction [20].

Calcined scales have been used as a catalyst for biodiesel synthesis [25]. In an exploration of the feasibility of converting waste rohu fish (*Labeo rohita*) scale into a high-performance, reusable, and low-cost heterogeneous catalyst for the synthesis of biodiesel from soybean oil, thermo-gravimetric analysis (TGA) and X-ray diffraction (XRD) analysis revealed that a significant portion of the main component of fish scale (i.e., hydroxyapatite) can be transformed into β-tri-calcium phosphate when calcined above 900 °C for two hours. Scanning electron microscopy morphology studies of the calcined scale depicted a fibrous layer with a porous structure [25].

Scale-supported Ni catalysis has also been developed for biodiesel synthesis [27]. A novel Ni–Ca–hydroxyapatite solid acid catalyst was prepared through wet impregnation of Ni(NO_3_)_2_·6H_2_O on pretreated waste fish scales. The efficacy of the developed catalyst, which possessed a specific surface area and catalyst acidity, was evaluated through esterification of the free fatty acids of pretreated waste soybean fry oil in a semibatch reactor [27].

#### 2.1.4. Skin

Skin contains approximately 30% collagen [37]. Skin from tilapia, carp, and redfish was treated by acid/alkali hydrolysis to produce collagen peptides [18,19,37]. Collagen from tilapia skin has been studied for biomedical applications [37]. Acid-soluble collagens (ASC) have been prepared from carp (*Cyprinus carpio*) skin, scale, and bone. The yields of skin ASC, scale ASC, and bone ASC are 41.3%, 1.35%, and 1.06% (on a dry weight basis), respectively [18]. Skin gelatin hydrolysate from tilapia has been produced using thermal hydrolysis with retorting treatment (at 121 °C for 30 min); the skin gelatin hydrolysate showed antioxidant activity [29]. Certain free amino acids and oligopeptides in hydrolysates of tilapia skin gelatin have been suggested to play an important role in their antioxidant properties [29].

### 2.2. Enzymatic Procedure

#### 2.2.1. Head and Frame

The preparation and characterization of fish protein hydrolysates from different species, enzymes, and hydrolysis conditions have been extensively studied [7]. Most fish protein hydrolysates come from the head and frame being treated with enzymatic procedures [4,5,6,7,8]. For instance, hydrolysates from horse mackerel treated with a mixture of subtilisin and trypsin showed antioxidant activity [5]. Defatted salmon backbone treated by enzyme hydrolysis produced hydrolysates that demonstrated antidiabetic and antihypertensive activities [6]. Waste material from *S. canicula* (small-spotted catshark) was hydrolyzed by commercial proteases (Alcalase, Esperase, and Protamex) to produce hydrolysates with antihypertensive and antioxidant activities [7]. Chondroitin sulfate has been produced from the head, skeleton, and fins of *S. canicula* by a combination of enzymatic, chemical precipitation, and ultrafiltration methodologies [8].

#### 2.2.2. Viscera

The viscera of catla (Indian carp) and Atlantic cod have been treated by Alcalase hydrolysis to produce fish food [9], microbial growth medium [10], and fish protein hydrolysates [11]. The enzymatic hydrolysates of Arctic cod viscera have been developed as a growth medium for lactic acid bacteria [12]. Nile tilapia viscera treated by Alcalase or intestinal hydrolysis have been investigated for the production of fish protein hydrolysates [13]. Sardine viscera also produce hydrolysates when treated with pepsin [14] and trypsin [15].

#### 2.2.3. Scales

Almost all studies on fish-scale reutilization have focused on the preparation of collagen peptides [21,22,23,24,28]. Sea bream scales hydrolyzed by protease produce collagen peptides [23]. Likewise, croaker scales treated by trypsin/pepsin hydrolysis have been found to produce antioxidant collagen peptides [28]. The antioxidant activities of the obtained three collagen peptides are due to the presence of hydrophobic amino acid residues within the peptide sequences [28].

The scales of four major cultivated fish in Taiwan, *Lates calcarifer*, *Mugil cephalus, Chanos chanos*, and *Oreochromis* spp., show Fe(II)-binding activity when hydrolyzed by papain and Flavourzyme [24]. Tilapia (*Oreochromis* sp.) scales were hydrolyzed by a given combination of proteases (1% Protease N and 0.5% Flavourzyme), and the obtained fish-scale collagen peptides (FSCPs) were shown to be able to effectively penetrate the stratum corneum to the epidermis and dermis [24,38]. Scales have also been used in scale-supported Ni catalysis during biodiesel synthesis [27].

#### 2.2.4. Skin

It has been suggested that the hydrolysis of salmon skin by bacterial protease produces antioxidant peptides [30]. Treatment of Alaskan pollock skin by Alcalase hydrolysis has been investigated for its production of antioxidant peptides [31]. The pepsin-soluble collagen obtained by hydrolyzing the skins of small-spotted catfish, blue sharks, swordfish, and yellowfin tuna with pepsin also shows antioxidant activity [32]. Collagen can be degraded much more easily than skin protein, but it commonly shows weaker antioxidant capability. The hydrolysate of salmon skin proteins prepared with bacterial extracellular proteases displays the strongest antioxidant activity. The amino acid composition of skin proteins is more complicated than that of collagen, the amino acids of skin proteins may contain more potential antioxidant peptide sequences [32,39].

### 2.3. Fermentation Procedure

*S. canicula* (small-spotted catshark) viscera have been used as a substrate to produce hyaluronic acid via *Streptococcus zooepidemicus* fermentation. This study investigated the production of hyaluronic acid by *Streptococcus equi* subsp. *zooepidemicus* in complex media formulated with peptones obtained from *S. canicula* viscera byproducts [16]. Scales have also been used to produce collagenase-like enzymes via microbial fermentation [26].

## 3. Use of Shrimp and Crab Processing Byproducts

The amount of shrimp and crab waste produced by the shellfish processing industry has dramatically increased in recent years. In addition to edible parts, the amount of chitin-containing waste can be as high as 60%–80% of the biomass. Shrimp and crab shells contain chitin, protein, and a high ratio of mineral salts. Chitin has a structure similar to cellulose and peptidoglycan and is the second most abundant biopolymer on earth next to cellulose [2].

Chitin has excellent properties, including biodegradability, biocompatibility, non-toxicity, and adsorption. Chitosan is a cationic polysaccharide obtained by either the *N*-deacetylation of chitin under alkaline conditions or enzymatic hydrolysis in the presence of a chitin deacetylase. Chitin, chitosan, and their derivatives have a number of industrial and medicinal applications, due to their antimicrobial and antioxidant activities, biocompatibility, biodegradability, antitumor activity, hemostatic activity, and antihypertensive and wound-healing properties [40,41,42,43,44,45,46,47,48,49,50,51,52,53,54,55,56,57,58].

Chitin is normally produced from shrimp and crab shells via chemical pretreatments of hot-alkali deproteinization and acid demineralization [2]. As such, most studies on the recycling of chitin-containing marine byproducts have focused on the preparation of chitin and its derivatives by chemical processes.

### 3.1. Chemical Procedures

Shrimp and crab shells must be demineralized and deproteinized to obtain chitin and chitosan [59,60,61,62,63,64,65,66]. Chitin and chitosan are commonly obtained from shrimp and crab shells using inorganic acids for demineralization and strong alkali for deproteinization. The harvested chitin, chitosan, and their derivatives have been investigated for their agricultural, food, environmental, fine chemical, and pharmaceutical applications [2]. Chemical treatments can produce purer chitin and chitosan than biological procedures; however, the waste materials from acid and alkali treatments contribute to environmental pollution and reduce the chitin quality. As such, chitin-containing waste could potentially become a precious bioresource if converted by biological processes to create high-value-added products [2,67,68,69,70,71,72,73,74,75,76,77,78,79,80,81,82,83,84,85,86,87,88,89,90] (Table 2).

### 3.2. Biological Procedures

Traditional biological treatments include enzymatic deproteinization by proteases and fermentation deproteinization by protease-producing bacteria (Table 2). The use of microbial proteolytic enzymes for the deproteinization of crustacean waste is a current trend in the conversion of waste into useful bioactive materials such as chitin [59,67,68,69,70,71,83], proteases [59,89,90], chitinases/chitosanases [86,88,89,91], chitin/chitosan oligomers [86,91], and α-glucosidase inhibitors [87,90]. The bioconversion procedure is a simple, inexpensive alternative to chemical methods employed in the preparation of chitin. To overcome the drawbacks of chemical procedures, studies have isolated many proteolytic and/or chitinolytic enzyme-producing bacteria using shrimp and crab shells as their sole carbon/nitrogen (C/N) source [2,72,73,74,75,76,77,78,79,80,81,82,83,84,85,86,87,88,89,90,91]. It is assumed that the shrimp and crab shells will be deproteinized by the protease produced by the bacteria during fermentation. Furthermore, the reclamation of chitin waste as the C/N source not only solves the environmental issue but reduces the production costs of bioconversion (Table 2).

In addition to chitin, shrimp waste also contains several bioactive compounds, such as astaxanthin, amino acids, and fatty acids [92,93,94,95,96,97,98,99,100,101]. These bioactive compounds have a wide range of applications, including those in the medical, therapeutic, cosmetic, paper, pulp, and textile industries, as well as in biotechnology and food [95,96,97,98,99,100,101,102,103,104]. Pacheco et al. [95] recovered chitin and astaxanthin from shrimp waste that was fermented using lactic acid bacteria, while Parjikolaei et al. [99] designed a green extraction method using sunflower oil to recover astaxanthin from shrimp waste. Amado et al. [96] reported on the recovery of high concentrations of astaxanthin by the ultrafiltration of wastewater used to cook shrimp and indicated that astaxanthin is associated with retained proteins that have a high molecular weight. Hydrolysates from these three protein-concentrated fractions showed very potent angiotensin-I-converting enzyme (ACE)-inhibitory and ß-carotene bleaching activities compared to hydrolysates from other fish and seafood species [96]. The extracted astaxanthin has been investigated as a possible food ingredient or color additive [100,101].

## 4. Use of Squid Processing Byproducts

Squid is an important commercial seafood worldwide. After processing, there are many byproducts and waste materials, including the heads, viscera, skin, and ink (Table 3). Many researchers have investigated the reclamation and potential use of these byproducts following different treatments [2,87,88,89,90,91,102,103,104,105,106,107,108,109,110,111,112,113,114,115,116,117,118,119,120,121,122,123,124,125,126,127,128,129,130,131,132,133]. For instance, chemical and/or biological processes were used to produce peptides with antioxidant activity from squid viscera autolysates [102]. Enzymatic hydrolysis of dried squid heads resulted in a high protein content with elevated levels of glutamic acid [103]. β-chitin was produced from squid pens following chemical and biological procedures [111,112]. Acid- and pepsin-soluble collagens were isolated from the outer skins of squid [104], and peptides with angiotensin-I-converting enzyme (ACE)-inhibitory and antihypertensive activities were produced from pepsin-hydrolysates of squid skin gelatin [105].

During squid pen fermentation, owing to the liquefaction of protein and chitin, a bioactive-material-rich liquor is formed, containing peptides, amino acids, chitooligomers, and other materials [2,59]. Most research on the recycling of squid pens has concentrated mainly on the chemical preparation of chitin and chitosan [110]. To further enhance utilization, the conversion of squid pens by microbial fermentation has recently been investigated for its production of bioactive materials (Table 3) [2]. Examples include the production of biofertilizers from squid pens by *Lactobacillus* subsp. *paracasei* fermentation [2], production of exopolysaccharides [114,115,116], biosurfactants [115], homogentisic acid [87,117], α-glucosidase inhibitors [87,118], chitosanases [88,89], and proteases [89] from squid pens by *Paenibacillus* fermentation [114,115,116], production of tyrosinase inhibitors and insecticidal materials from squid pens by *Burkholderia cepacia* [119], and production of chitosanases [120] and protease with anti-α-glucosidase activity [90] from squid pens by *Bacillus* fermentation.

### 4.1. Squid Viscera/Heads/Skin

It is estimated that more than 40% of the total body weight of squid ends up as processing byproducts, including the viscera, pens, and skins. The major component in these byproducts is protein, which may be hydrolyzed by enzymes or acid to generate peptides and free amino acids. Acid hydrolysis causes the destruction of hydrolysates and the formation of NaCl following neutralization, which can make the end product unpalatable. However, both autolysis by protease present in squid viscera and enzymatic hydrolysis produce fewer undesirable byproducts [108]. Biologically hydrolyzed products demonstrate antioxidant activity [102], contain collagen [102] and amino acids with umami [103], are used in fish sauce [105], and show growth-promoting and attractant properties in fish culture [134]. There have also been reports about the extraction of protease [108], squid oil, and squid fat [109] from viscera (Table 3).

Squid skin is an excellent source of collagen and is used in the manufacturing of cosmetics [104,105]. Collagen-based biomaterials have been widely used due to their binding capabilities. However, the properties and potential uses of new collagen sources are still under investigation. Squid collagen was investigated as a potential plasticizer in the preparation of biofilms in combination with chitosan [135]. The chitosan/collagen (85/15) blend produced a transparent and brittle film with a high percentage of elongation at the break, and low tensile strength in comparison to chitosan films [135]. Due to the anti-bacteriostatic properties of chitosan and the cellular functions of collagen, chitosan/collagen blend biofilms may have the potential to be used as a wound dressing [135,136]. Similar results were also reported when cartilaginous fish collagen was used in combination with chitosan to produce a composite film. When compared to collagen films, the chitosan/collagen blend film showed lower water solubility and lightness [137]. The chitosan/collagen-based biofilm has potential UV barrier properties and antioxidant activity, and they could possibly be used as a green bioactive film to preserve nutraceutical products [137].

Squid tentacles have suckers which allow them to adhere to surfaces and move the organism. The structural, mechanical, and bioprocessing strategies of the biological systems involved in squid sucker rings have recently been investigated in order to develop environmentally benign ways to synthesize novel materials for biomedical and engineering applications [138,139,140,141].

### 4.2. Squid Pens

Unlike shrimp and crab shells (both major sources of α-chitin), squid pens are a rich source of β-chitin and contain low amounts of inorganic compounds [2,142]. Squid pens contain protein (61%), chitin (38%), and trace amounts of mineral salts [2,134]. The major product from squid pens is β-chitin, which is normally prepared via chemical processes [111,112] or a combination of chemical and enzymatic procedures [104]. Such β-chitin preparations are further modified by chemical, physical, and/or biological procedures to improve their properties. As shown in Table 3, squid pens are valuable as a starting material in the preparation of β-chitin [111,112] and the production of bioactive compounds via bioconversion with microbial fermentation [2,114,115,116,117,118,119,120,121,122,123,142] and enzymatic hydrolysis [110]. The bioactive materials obtained include biofertilizers and proteases by *Lactobacillus* fermentation [2]; exopolysaccharides [114,115,116], biosurfactants [114,115,116], homogentisic acid [87,117], tryptophan [117], α-glucosidase inhibitors [87,118] by *Paenibacillus* fermentation; chitosanases [88,89] and proteases [89] by *Paenibacillus* fermentation; tyrosinase inhibitors by *Burkholderia* fermentation [119]; chitosanases [120], chitooligomers [120], and protease with anti-α-glucosidase activity by *Bacillus* [90], chitosanases and chitooligomers [121] by *Penicillium*; and chitinases [91] and chitin oligomers [91] by *Streptomyces*.

### 4.3. Squid Ink

Among the components of squid ink, melanin has received the most interest and has been used in comparative studies of melanogenesis. Squid ink melanin is the most commonly used melanin. The ink is a mixture of secretions from the ink sac, including melanin, glycosaminoglycan-like polysaccharides, enzymes, proteins, and lipids [125,126,127,128,129,130,131,132,133]. Melanin is the main component, resulting in its dark color. As shown in Table 3, recent medical investigations suggest that squid ink is a multifunctional bioactive marine drug that has antioxidative [125,126], anti-inflammatory [125], anti-neoplastic [127], antitumor [128], antihypertensive [129], anti-radiation, antimicrobial, and anticoagulant activities, as well as the ability to protect against testicular damage [143].

## 5. Conclusions

Globally, fish, shrimp, crab, and squid are some of the most important commercial marine resources. Processing the byproducts of these organisms provides rich sources of proteins, lipids, and chitin. The reclamation of these components via chemical, physical, and biological procedures can aid in solving the environmental problems associated with cost of other bioactive materials, such as enzymes, antioxidants, antidiabetic materials, and exopolysaccharides. If these issues are dealt with in a serious and continuous manner, the costs of fishery processing should not pose a problem.

## Figures and Tables

**Table 1:** Use of fish processing byproducts.

Byproduct	Treatment	Product (Application)	Reference
**Fish head and frame**			
Kingfish head and frame	Enzyme	Fish protein hydrolysate	[4]
	Microwave		
	Chemical		
Horse mackerel discard	Subtilisin and trypsin	Fish protein hydrolysate (antioxidant)	[5]
Defatted salmon backbone	Enzyme hydrolysis	Fish protein hydrolysate (antidiabetic and antihypertensive)	[6]
Catshark discard	Alcalase, Esperase, and Protamex	Fish protein hydrolysate (antihypertensive and antioxidant)	[7]
Catshark head and frame	Enzyme	Chondroitin sulfate	[8]
	Chemical		
	Physical		
**Fish viscera**			
Catla viscera	Alcalase	Fish diet	[9]
Atlantic cod viscera	Alcalase	Microbial growth medium	[10]
Atlantic cod viscera	Alcalase	Fish protein hydrolysate	[11]
Arctic cod viscera		Microbial growth medium	[12]
Nile tilapia viscera	Alcalase	Fish protein hydrolysate	[13]
Nile tilapia viscera	Intestinal	Fish protein hydrolysate	[13]
Sardine viscera	Pepsin	Fish protein hydrolysate	[14]
Sardine viscera	Trypsin	Fish protein hydrolysate	[15]
Catshark viscera	Conversion by *Streptococcus*	Hyaluronic acid	[16]
Catshark pancreas	Purification	Trypsin	[17]
**Fish scales**			
Carp skin, scale, and bone	Acid/alkali hydrolysis	Collagen peptides	[18]
Redfish skin, scale, and bone	Acid/alkali hydrolysis	Collagen peptides	[19]
Nile tilapia scale	Acid/alkali hydrolysis	Metallic ion removal	[20]
Snakehead scale	Protease hydrolysis	Collagen peptides	[21]
Fish scale	Protease hydrolysis	Collagen peptides	[22]
Sea bream scale	Protease hydrolysis	Collagen peptides	[23]
Fish scale	Papain and Flavourzyme	Collagen peptides	[24]
Fish scale	Calcination	Catalysis for biodiesel synthesis	[25]
Scale	Conversion by actinomycetes	Collagenase-like enzymes	[26]
Scale	Scale-supported Ni catalysis	Biodiesel synthesis	[27]
Croaker scale	Trypsin/pepsin hydrolysis	Antioxidant collagen peptides	[28]
**Fish skin**			
Tilapia skin	Thermal hydrolysis	Antioxidant gelatin hydrolysates	[29]
Salmon skin	Bacterial proteases hydrolysis	Antioxidant peptides	[30]
Alaskan pollock skin	Alcalase	Antioxidant peptides	[31]
Shark, swordfish, and tuna skin	Pepsin	Antioxidant peptides	[32]

**Table 2:** Use of shrimp and crab processing byproducts.

Byproduct	Treatment	Product (Application)	Reference
Shrimp shells	Enzymatic/chemical	Chitin, chitosan	[60]
Shrimp shells	Enzymatic/chemical	Chitin	[61]
Shrimp shells	Chemical	Chitin, chitosan	[62,63,64,65]
Shrimp shells	*Bacillus* protease	Chitin	[67]
Shrimp shells	*Paracoccus* protease	Chitin	[68]
Shrimp shells	*Pseudomonas* protease	Chitin	[69,70]
Shrimp shells	Crab viscera protease	Chitin	[71]
Shrimp shells	*Bacillus*	Chitin	[72,73,74,75]
Shrimp shells	Lactic acid bacteria	Chitin	[76,77]
Shrimp shells	Lactic acid bacteria	Chitin, carotenoid	[78]
Shrimp shells	Lactic acid bacteria	Chitin	[79]
Shrimp shells	*Lactobacillus*/*Serratia*/*Rhizopus*	Chitin	[80]
Shrimp shells	*Lactobacillus*/*Serratia*	Chitin	[81]
Shrimp shells	Conversion by *Bacillus*	Chitin	[2]
Shrimp shells	Conversion by *Chryseobacterium*	Chitinase, protease	[2]
Shrimp shells	Conversion by *Pseudomonas*	Chitinase, protease	[2]
Shrimp shells	Conversion by *Paenibacillus*	α-glucosidase inhibitors	[82]
Shrimp shells	Conversion by *Serratia*	Chitinase, protease	[2]
Crab shells	Conversion by *Vibrio*	Protease	[2]
Crab shells	Crab viscera protease	Chitin	[71]
Crab shells	Conversion by *Pseudomonas*	Chitin	[83]
Lobster shells	Conversion by *Paenibacillus*	α-glucosidase inhibitors	[82]
Shrimp heads	Chemical	Chitin	[66]
Shrimp heads	Autolysis	Protein hydrolysate	[92]
Shrimp heads	Chemical process	Chitin, glycosaminoglycan	[92]
Shrimp heads	Ethanol extraction	Carotenoid	[92]
Shrimp heads	Conversion by *Bacillus/Rhizobium*	α-glucosidase inhibitors	[84]
Shrimp heads	Conversion by *Brevibacillus*	Protease, chitin, chitin oligomers	[59]
Shrimp heads	Conversion by *Paenibacillus*	α-glucosidase inhibitors	[85]
Shrimp waste	Conversion by *Paenibacillus*	Chitosanase, chitosan oligomers	[86]
Shrimp waste	Autolysis	Chitin	[93]
Shrimp waste	Oil extraction	Astaxanthin	[94]
Shrimp cooking wastewater	Lactic acid bacteria	Chitin, astaxanthin	[95]
Shrimp waste	Ultrafiltration/hydrolysis	Astaxanthin/bioactive peptides	[96]
Shrimp waste	Sunflower oil extraction	Astaxanthin	[99]
Shrimp waste	Lipid extraction	Astaxanthin, fatty acids, α-tocopherol	[100]
Shrimp waste	Lipid extraction	Astaxanthin	[101]

**Table 3:** Use of squid processing byproducts.

Byproduct	Treatment	Product (Application)	Reference
**Squid viscera/head/skin**			
Viscera	Autolysis	Antioxidant peptides	[102]
Head	Enzymatic hydrolysis	Sweet, umami amino acids	[103]
Skin	Acid/pepsin soluble	Collagen	[104]
Skin	Enzymatic hydrolysis	Gelatin hydrolysates	[105]
Viscera/head/skin	Endogenous proteases	Squid hydrolysate	[106]
Whole byproducts	Fast fermentation	Low-salt fish sauce	[107]
Squid hepatopancreas	Extraction	Carboxypeptidase	[108]
Squid viscera	Subcritical water hydrolysis	Squid oil and fat	[109]
**Squid pens**			
Squid pens	Chemical	Chitin/chitosan	[110,111,112]
	Enzymatic		
Squid pens	Chemical modification	Antioxidant *N*-carboxyethylated derivatives	[113]
Squid pens	Conversion by lactic acid bacteria	Biofertilizers, proteases	[2]
Squid pens	Conversion by *Paenibacillus*	Exopolysaccharides, biosurfactants	[114,115,116]
Squid pens	Conversion by *Paenibacillus*	Homogentisic acid, tryptophan	[117]
Squid pens	Conversion by *Paenibacillus*	α-Glucosidase inhibitors	[118]
Squid pens	Conversion by *Paenibacillus*	α-Glucosidase inhibitors, homogentisic acid	[87]
Squid pens	Conversion by *Paenibacillus*	Chitosanases	[88]
Squid pens	Conversion by *Paenibacillus*	Chitosanases, proteases	[89,90]
Squid pens	Conversion by *Burkholderia*	Tyrosinase inhibitors	[119]
Squid pens	Conversion by *Bacillus*	Chitosanases, chitooligomers	[120]
Squid pens	Chemical	Chitin	[121]
Squid pens	Chemical	Chitosan	[122]
Squid pens	Conversion by *Penicillium*	Chitosanases, chitooligomers	[123]
Squid pens	Conversion by *Streptomyces*	Chitinases; chitin oligomers	[91]
Chitosan extraction effluent	Protease hydrolysis	Antioxidant peptides	[124]
**Squid ink**			
Squid ink	Non treatment	Antioxidant, anti-inflammation	[125,126]
Squid ink	Non treatment	Anti-neoplastic	[127]
Squid ink	Chemical	Antitumor	[128]
Squid ink	Chemical	Antihypertensive	[129]
Squid ink	Chemical	Functional food	[130,131]
Squid ink	Squid ink polysaccharide-chitosan	Wound-healing sponge	[132]
Squid ink	Squid ink melanin-Fe	Iron deficiency anemia	[133]
